# PLGA-Based In Situ-Forming Implants, a Quality by Design Perspective

**DOI:** 10.3390/pharmaceutics18030351

**Published:** 2026-03-12

**Authors:** Nayelli Campos-Morales, Luz Graciela Cervantes-Pérez, Alicia Sánchez-Mendoza, María Sánchez-Aguilar, José Juan Escobar-Chávez, Lizbeth Martínez-Acevedo, Moises Job Galindo-Pérez, Jorge Esteban Miranda-Calderon

**Affiliations:** 1 Ciencias Farmacéuticas, Universidad Autónoma Metropolitana, Unidad Xochimilco, Calzada del Hueso 1100, Colonia Villa Quietud, Alcaldía Coyoacán, Mexico City C.P. 04960, Mexico; 2 Instituto Nacional de Cardiología, Juan Badiano 1, Belisario Domínguez Secc 16, Tlalpan, Mexico City C.P. 14080, Mexico; 3 Unidad de Investigación Multidisciplinaria-L 12, Facultad de Estudios Superiores Cuautitlán, Universidad Nacional Autónoma de México, Carretera Cuautitlán-Teoloyucan, Km 2.5 San Sebastián Xhala, Cuautitlán Izcalli C.P. 54714, Estado de Mexico, Mexico; 4 Departamento de Sistemas Biológicos, Universidad Autónoma Metropolitana, Unidad Xochimilco, Calzada del Hueso 1100, Colonia Villa Quietud, Alcaldía Coyoacán, Mexico City C.P. 04960, Mexico; 5 Departamento de Procesos y Tecnología, Universidad Autónoma Metropolitana, Unidad Cuajimalpa, Av. Vasco de Quiroga 4871, Santa Fe Cuajimalpa, Mexico City C.P. 05348, Mexico

**Keywords:** QbD, implant, injectable

## Abstract

In situ-forming implants (ISFIs) based on poly(lactic-co-glycolic acid) (PLGA) offer a promising platform for long-acting parenteral drug delivery, enabling minimally invasive administration without surgical implantation. However, the development and clinical translation of PLGA-based ISFIs are hindered by formulation complexity, sensitivity to aterial variability, and limited predictability of drug release, particularly during early implant formation. Although previous reviews have described formulation components and release mechanisms, a comprehensive integration of Quality by Design (QbD) principles with a focus on risk prioritization remains absent. This review examines the application of QbD to solvent-exchange PLGA-based ISFIs, with an emphasis on identifying critical material attributes (CMAs) governing implant formation, burst release, and long-term release performance. Risk-based prioritization of CMAs and the role of design of experiments are systematically discussed. Special attention is given to burst release as a major CMA affecting safety, efficacy, and translational robustness. The evidence indicates that formulation-driven CMAs, such as polymer physicochemical properties, drug characteristics, and solvent selection, exert a greater influence on ISFI performance than process-related parameters. This review provides a structured perspective to support rational formulation design, improved reproducibility, and enhanced clinical translation of PLGA-based ISFI systems.

## 1. Introduction

**Figure 1:**
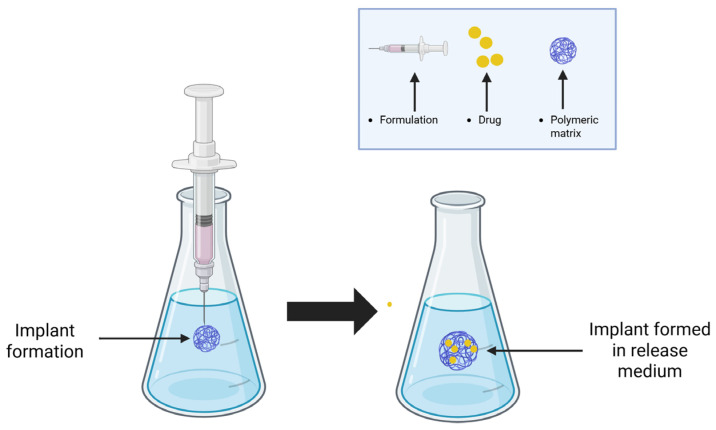
In situ-forming implants. Formation by solvent exchange. Created in BioRender. Campos, 2. (2025) https://BioRender.com/ow0ke2a, accessed on 10 December 2025.

In situ-forming implants (ISFIs) present an innovative technology that enables prolonged drug release without requiring surgical implantation [1]. ISFIs are defined by Dunn et al. as polymeric solutions administered parenterally using a fine-gauge needle, and they solidify upon injection by precipitating at the site [2] (Figure 1). The resulting structure, in response to the conditions, serves as a drug reservoir, facilitating controlled release [3,4,5].

ISFIs are minimally invasive and can conform to the shape of the target tissue, organ, or cavity [6], making them particularly suitable for administration in hard-to-reach anatomical sites [7]. Their applications can be designed for both human and veterinary use [8]. Several marketed pharmaceutical products are based on this technology, including formulations incorporating Atrigel^®^ technology™, such as Eligard^®^ for advanced prostate cancer, Atridox^®^ for periodontitis treatment, and Atrisorb^®^ and Atrisorb D^®^ for tissue regeneration [9,10]. The commercial product Eligard^®^ uses different PLGA copolymer ratios depending on the dose strength. The 7.5 mg formulation contains PLGA 50:50, whereas the 22.5 mg and 30 mg versions incorporate PLGA 75:25, and the 45 mg product employs PLGA 85:15. According to publicly available prescribing information, the reconstituted formulation contains approximately 45–50% (*w*/*w*) PLGA and 50–55% (*w*/*w*) N-methyl-2-pyrrolidone (NMP), with leuprolide acetate representing approximately 2–4% (*w*/*w*) of the total formulation, depending on the dose strength [11].

Atridox^®^ contains doxycycline hyclate formulated with 36.7% (*w*/*w*) poly(DL-lactide) and 63.3% (*w*/*w*) N-methyl-2-pyrrolidone (NMP), forming an implant upon contact with gingival fluid following injection into the periodontal pocket [12]. Likewise, Atrisorb^®^ and Atrisorb D^®^ consist of poly(DL-lactide) (PDLLA) dissolved in N-methyl-2-pyrrolidone; their proportions are 37% and 63% respectively. The active compound (doxycycline hyclate) accounts for approximately 10% (*w*/*w*) of the total formulation [13].

Despite their clinical and technological advantages, ISFI performance is highly sensitive to formulation composition and process-related variables. Polymer properties, solvent selection, drug physicochemical characteristics, and implant formation dynamics strongly influence critical attributes such as injectability, implant morphology, solvent exchange, and especially the initial burst release [14]. This intrinsic complexity often leads to high batch-to-batch variability and unpredictability of in vivo performance, representing a major challenge for formulation development and clinical translation [15].

Quality by Design (QbD) offers a systematic framework to address these limitations by linking predefined product objectives with a scientific understanding of critical material attributes (CMAs) and critical quality attributes (CQAs) and identifying potential risks that may impact quality, allowing for proactive control [16,17]

Additionally, the knowledge gained about the variables and parameters affecting the process helps reduce the likelihood of batch rejection, minimizes variability, and decreases defects in final products [12].

In this context, the present review focuses on PLGA-based ISFIs formed by solvent exchange mechanisms, providing a structured analysis of formulation components, implant formation, drug release behavior, and risk-based development strategies under a QbD paradigm. This approach aims to support the rational design, optimization, and reproducibility of ISFI systems intended for long-acting parenteral drug delivery.

## 2. Quality by Design (QbD) Implementation of ISFIs

Quality by Design (QbD) provides a structured and systematic framework [18] to guide the development of ISFIs by identifying, prioritizing, and controlling the variables that govern product performance, safety, and reproducibility. In contrast to empirical trial-and-error approaches, QbD emphasizes a risk-based understanding of how formulation composition and process parameters influence critical quality attributes (CQAs) throughout the product lifecycle.

In PLGA-based ISFIs, the application of QbD is particularly relevant due to the inherent complexity of solvent-exchange systems, where small variations in material attributes can result in significant changes in implant formation, drug release behavior, and batch-to-batch consistency [19]. Zhang and collaborators discussed the implementation of QbD in the production of long-acting PLGA/PLA-based microspheres for the therapeutic peptide and protein drug delivery. Their study included the definition of the quality target product profile (QTPP), defined CQAs, CMAs and CPPs, and the establishment of a control strategy. They concluded that the QbD approach enables guiding the systematic development of drug products [20]. The QbD system involves a series of steps that complement each other, some of which are listed below:

1. Define the quality target product profile (QTPP).
2. Understand the critical quality attributes (CQAs) of the target product.
3. Identify critical material attributes (CMAs).
4. Identify critical process parameters (CPPs).
5. Establish the design space and control strategy.
6. Continuous verification [21].

Within this framework, ISFI development is driven by the definition of the quality target product profile (QTPP), followed by the identification of critical quality attributes (CQAs), critical material attributes (CMAs), and critical process parameters (CPPs) [22]. The following sections describe the stepwise application of QbD to PLGA-based solvent-exchange ISFIs.

### 2.1. Define the Quality Target Product Profile (QTPP)

**Figure 2:**
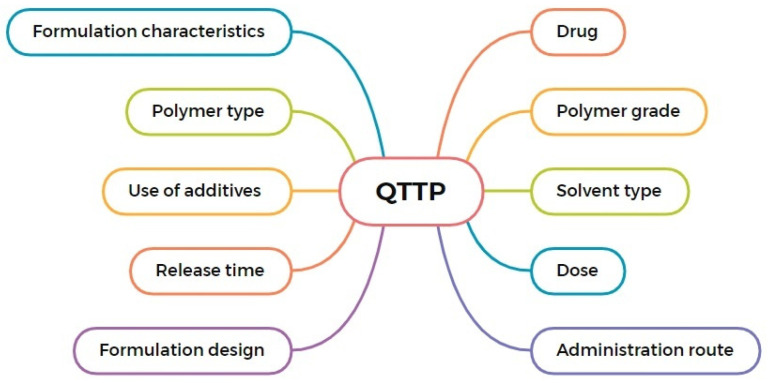
Key considerations for designing a QTPP for ISFI formulations.

The quality target product profile (QTPP) defines the intended clinical performance and quality characteristics that an ISFI formulation must achieve [23]. For PLGA-based ISFIs, the QTPP establishes key constraints related to dosage form, route of administration, drug loading, release duration, and safety requirements, thereby guiding all subsequent formulation and development decisions [16]. Figure 2 shows key elements to consider when designing a QTPP for ISFI formulations, highlighting the interplay between formulation characteristics and product performance.

Importantly, the definition of the QTPP enables early identification of attributes that may pose risks to product performance or patient safety, allowing these risks to be addressed proactively during formulation design [24]. In solvent-exchange ISFIs, parameters such as injectability, implant formation behavior, and release duration are particularly critical, as they directly influence therapeutic efficacy and local tolerability.

In addition, Table 1 presents a practical application of the QTPP concept based on a previously reported PLGA-based ISFI system, illustrating how predefined quality objectives serve as the foundation for identifying critical quality attributes in later stages of QbD implementation. This table is based on research conducted by Patel and collaborators, who mention the use of ISFIs in post-ablative adjuvant treatment with fluorescein in rats, evaluating the impact of different PLGA molecular weights and how the release medium influences implant behavior [25].

### 2.2. Critical Quality Attributes (CQAs) of the Target Product

CQAs are the physicochemical characteristics and identification tests that ensure the product’s quality and efficiency [11,26]. These characteristics must be defined by value or a range; they can be specifications or attributes [27]. Sheshala and colleagues presented a study to further explain this step. They developed an ISFI system for the sustained delivery of triamcinolone acetonide and conducted physicochemical characterization, including viscosity measurements, dynamic viscoelastic analysis, and syringeability test. They concluded that viscosity increases with higher drug concentration and PLGA formulations containing a higher polymer grade (greater L/G ratio) [28].

A simple way to determine whether an attribute could compromise the final product’s quality is by answering two key questions:

- Will this attribute change the formulation or process in a way that affects the final product quality?
- Does the failure of this attribute compromise efficacy and safety? [29]

**Figure 3:**
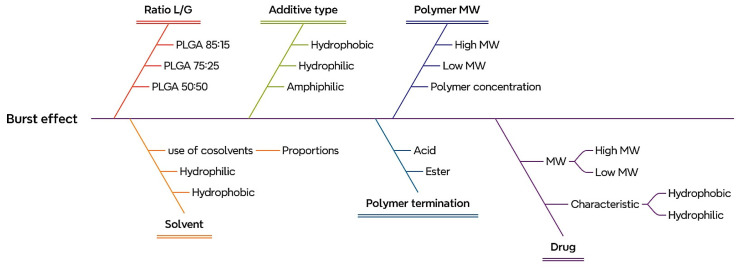
Ishikawa diagram for risk identification of CQAs and formulation variables affecting the initial burst release of PLGA-based ISFIs.

The evaluation of these questions will facilitate the identification of CQAs in the process under assessment [30]. These attributes are generally related to the appropriate selection of excipients (additives and polymers), drugs, and their optimal quantities [31]. The application of this stage of the process for in situ-forming implants is illustrated in Figure 3. The application of this risk identification stage within the QbD framework for in situ-forming implants is illustrated using an Ishikawa (fishbone) diagram, focusing on factors potentially contributing to the initial burst release.

In PLGA-based ISFIs, an initial burst release is one of the most critical CQAs, as it can lead to undesirably high drug levels immediately after administration, compromising safety and therapeutic performance [13]. This analysis provides a better understanding of the variables to consider for controlling this effect. Table 2 presents an applied example for an ISFI, outlining the potential attributes to be evaluated in ISFI formulation and assessing whether the parameters are truly critical, as previously discussed.

#### 2.2.1. Release of the Drug in ISFI-Related CQAs

Drug release from ISFIs constitutes a key CQA and is governed by a combination of formulation composition. Drug release depends on several factors, including implant morphology, drug properties, formulation composition, the presence of additives, polymer type, and preparation techniques [32,33]. A typical release profile in these systems follows a triphasic pattern: initial release (burst effect), diffusion-mediated release, and polymer degradation [34,35].

**Figure 4:**
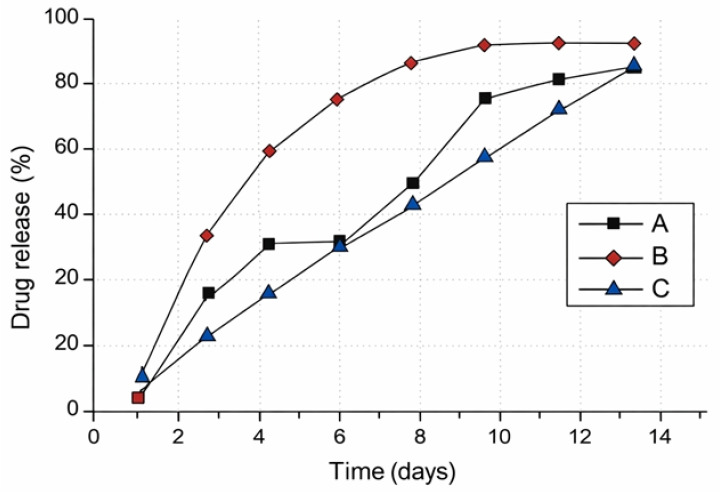
Potential release profiles in ISFIs. A. Triphasic release profile. B. Biphasic release profile. C. Zero-order release profile.

From a QbD perspective, the initial burst release represents the highest-risk phase of drug delivery, as excessive early release may lead to undesired systemic exposure or local toxicity [36]. Consequently, controlling burst release requires a holistic understanding of formulation-related CMAs rather than the isolated optimization of individual variables. For a clearer description of this release profile, Figure 4 illustrates the different potential release patterns of the drug in ISFI systems.

The release profile primarily depends on the characteristics of the drug and also on the polymer [37]. The study conducted by Suh and collaborators highlights the significance of this aspect. They evaluated the impact of altering the implant formation process by comparing five methods: direct injection into the medium, rapid freezing, gelatin capsules, dialysis, and PVA (polyvinyl alcohol) films. They assessed solvent diffusion into the surrounding medium, implant dimensions and release kinetics. Their findings indicated that direct injection into the medium, rapid freezing and gelatin capsules present a biphasic release profile, whereas dialysis and PVA films display a triphasic release profile. This difference is attributed to the fact that the first three methods result in a high burst release followed by system rupture, whereas the last two methods present a lower burst effect and a more pronounced lag phase [38]. The rate of drug release depends on parameters that are relatively easy to modify. Another example of how polymer degradation and matrix erosion are affected includes variables such as polymer grade or the combination within the formulation [39].

These observations highlight the inherent challenge of achieving reproducible release behavior in ISFIs and underscore the importance of incorporating release performance as a central focus of QbD-driven development.

#### 2.2.2. In Vitro Evaluation of ISFI-Related CQAs

In vitro evaluation is essential for characterizing ISFI implant formation and drug release; however, its value depends on whether the selected method is discriminatory, reproducible, and clinically relevant. A key limitation is that many in vitro tests unintentionally alter the phase inversion kinetics, implant geometry, and mass-transfer boundary conditions, thereby changing the magnitude of burst release and the subsequent release phases [40]. However, the drug release behavior observed in vitro does not necessarily reflect the release profile observed in vivo [25]. Therefore, in vitro methods should be interpreted primarily as comparative screening tools for ranking formulations and identifying high-risk CMAs, rather than as direct surrogates of clinical performance [41,42]. Several experimental approaches have been developed to evaluate ISFIs in vitro, each with distinct advantages and limitations:

Medium injection: In this widely used method, the liquid ISFI formulation is injected directly into an aqueous medium under controlled agitation and temperature, allowing the implant to form freely in an unconfined environment. However, the absence of mechanical constraints tends to exaggerate burst release, since solvent efflux and drug diffusion proceed without the resistive forces present in tissue. In response to these limitations, adapter-based approaches have been proposed to improve the standardization of injection geometry and sampling conditions, thereby reducing experimental artifacts and improving inter-laboratory reproducibility [43].

Flash freezing: In flash-freezing approaches, the injected ISFI is rapidly quenched in liquid nitrogen or a cryogenic medium to lock in the early implant structure prior to release testing or morphological analysis. This method is useful for dissecting the initial stages of phase inversion and drug distribution. However, the resulting implants often display irregular geometries and microstructural artifacts that can further enhance apparent burst release during subsequent incubation. Because freezing avoids the solvent-exchange process that occurs in vivo, its use should be explicitly justified (e.g., for comparative screening only), and results should not be used alone to inform design-space decisions [44].

Gelatin capsules: Encapsulation of the liquid formulation in capsules prior to immersion in the release medium provides geometric confinement during solvent exchange and depot formation. Consequently, the resulting release kinetics may reflect capsule-mediated mass transfer rather than formulation-driven solvent exchange [45].

Dialysis: Dialysis devices such as Float-A-Lyzer^®^ and Slide-A-Lyzer^®^ MINI allow the implant to form inside a compartment separated from the bulk medium by a semi-permeable membrane, through which the drug diffuses for sampling without direct physical disturbance of the depot. These systems introduce a controlled diffusional barrier, which attenuates apparent burst release and stabilizes long-term sampling. However, membrane resistance and potential drug–membrane adsorption may systematically underestimate true release rates, particularly for macromolecules and hydrophobic APIs. Some studies have demonstrated that dynamic dialysis configurations improve the correlation between in vitro and in vivo release behavior of PLGA ISFIs by reducing stagnant boundary layers and better simulating physiological exchange conditions. Similarly, recent efforts toward establishing IVIVC models for in situ-forming implants emphasize the necessity of controlling hydrodynamics and sink conditions to enhance translational predictability [44].

PVA films: PVA film or bag systems allow implant formation within a confined, water-soluble enclosure that regulates depot dimensions and solvent exchange gradients. By limiting rapid expansion and controlling surface area exposure, these systems often reduce burst release magnitude. A PVA-adapter method was specifically designed for PLGA ISFIs, demonstrating improved intra- and inter-laboratory reproducibility compared to direct injection and showing that it could distinguish formulation differences masked by uncontrolled implant geometry in conventional setups [46].

Despite these advancements, the most widely used method for ISFI formation remains direct injection into the release medium, a technique widely used in experimental ISFI studies. Establishing robust IVIVC for PLGA-based ISFIs requires integrating advanced in vitro models with mechanistic mathematical modeling and in vivo imaging strategies, as no current single method provides a reliable, universal surrogate for clinical depot performance. From a QbD perspective, in vitro release testing should not be viewed solely as a characterization tool, but as a risk-assessment strategy aimed at identifying formulation-dependent variability and ranking CMAs according to their impact on release behavior.

### 2.3. Identify Critical Material Attributes (CMAs) Related to Material Characteristics

The identification of CMAs constitutes a central step in the application of QbD to ISFI formulations, as material properties are the primary drivers of product performance and variability, such as excipients (surfactants or polymers) [47], as well as active pharmaceutical ingredients. Arévalo-Pérez and collaborators (2024) evaluated the QbD approach for designing a colon-targeted drug delivery system for diverticular disease treatment. By identifying CMAs and CPPs, they developed a comprehensive risk management framework [48].

The formulations of in situ-forming implants are relatively simple, as they consist of a limited number of materials, as shown in Table 3. It is important to note that this example is focused on a solvent exchange formation method using PLGA as a polymer.

#### 2.3.1. Drug-Related CMAs

From a QbD perspective, the drug represents a critical material attribute (CMA) in ISFI formulations, as its physicochemical properties strongly influence implant formation, drug release kinetics, and overall product performance. Key drug-related attributes include molecular weight, lipophilicity (Log P), solubility and potential interactions with the polymer matrix.

**Figure 5:**
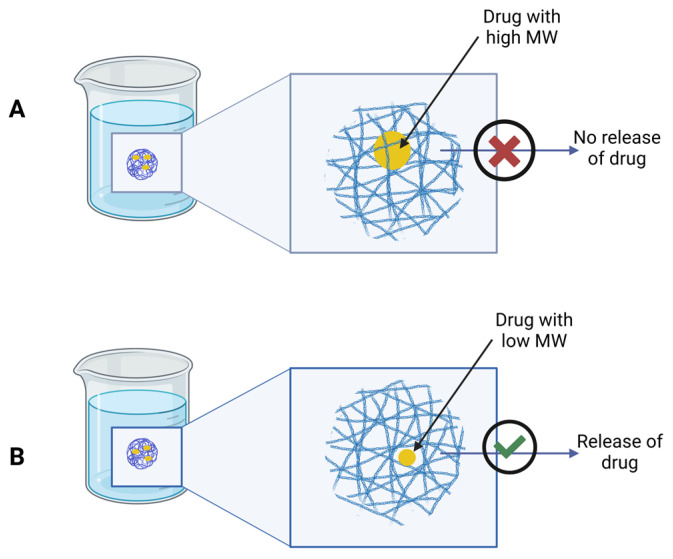
Representation of drug release into the medium with molecules of different molecular weights: (**A**) Small molecule. (**B**) Larger molecule. Created in BioRender. Campos, 2. (2025) https://BioRender.com/7c2n0g0, accessed on 10 December 2025.

Molecular size modulates drug diffusion within the formed implant. Low-molecular-weight drugs diffuse more readily through the polymer network, resulting in faster release rates, whereas larger molecules experience steric hindrance, leading to prolonged retention within the depot [40] (Figure 5). Small-molecule excipients can also act as matrix-forming components in in situ gelling systems. Recent research indicates that small-molecule gelators, such as long-chain fatty acids and certain thermoresponsive polymers, can serve as matrix-forming agents. For example, Su et al. (2022) reviewed how small molecular gels form via self-assembly and modulate drug release behavior in injectable depots [6].

Lipophilicity is another relevant CMA, as ISFIs are commonly administered via subcutaneous or intramuscular routes and are often formulated using hydrophobic polymers such as poly (lactic-co-glycolic acid) (PLGA) [49]. Drugs with higher Log P values generally exhibit improved compatibility with the polymer matrix, facilitating sustained release, whereas poorly compatible drugs may lead to heterogeneous drug distribution and variable release kinetics [50,51].

Drug solubility plays a central role in determining the release profile of ISFIs [52], as does the solvent used. In addition, drug–polymer interactions, particularly with PLGA terminal groups, can significantly affect release behavior. Differences between acid-terminated and ester-terminated PLGA have been shown to alter drug–polymer affinity and, consequently, the duration and consistency of drug release [53,54]. Additionally, the selected polymer must exhibit adequate physicochemical stability as well as biocompatibility [28,55].

Therefore, drug selection and characterization are critical steps within a QbD framework to ensure predictable performance and reproducibility of ISFI formulations.

The available evidence demonstrates that drug-related physicochemical properties play a decisive role during the early stages of ISFI formation and subsequent release behavior. Molecular weight, lipophilicity, and aqueous solubility not only modulate diffusion through the polymer matrix but also strongly influence drug distribution during phase inversion, thereby affecting burst release magnitude and reproducibility. Importantly, these effects are not independent of the formulation environment, as drug–polymer affinity and solvent-mediated partitioning further amplify variability. From a QbD perspective, these findings underscore the need to prioritize drug-related CMAs early in development, as inadequate alignment between drug properties and formulation design may compromise both safety and translational robustness, regardless of downstream process optimization.

#### 2.3.2. Polymer-Related CMAs

From a QbD perspective, the polymer constitutes a CMA with a dominant influence on implant formation, drug release kinetics, and batch-to-batch reproducibility of ISFIs.

PLGA is the most widely used due to its established biocompatibility, regulatory acceptance, and tunable physicochemical properties [56,57].

Key PLGA attributes that must be controlled within a QbD framework include the lactide-to-glycolide (L/G) ratio, molecular weight and end-group termination [58]. Variations in the L/G ratio directly modulate polymer hydrophilicity and degradation rate, thereby influencing depot residence time and drug release duration [59]. A higher GA content compared to LA results in a faster degradation rate and lower hydrophobicity [60,61].

Polymer molecular weight is another critical determinant of ISFI performance. Wang and collaborators provided evidence of the impact of PLGA properties, such as molecular weight (18–31 kDa) and L/G ratio (75:25 or 85:15), in formulations of naproxen, meloxicam and risperidone. It was shown that a higher PLGA molecular weight, from 18 kDa to 25 kDa, resulted in a longer release duration [62]. Another example of this is the study conducted by Patel and collaborators, who examined the effect of different PLGA molecular weights (PLGA 50:50 of 18, 33, and 50 kDa) on the release of fluorescein in vivo and in vitro experiments. Their findings revealed that lower molecular weight polymers exhibit a reduced burst effect (0–24 h) showing a more pronounced difference during this period compared to higher molecular weight polymers [25]. These relationships highlight the need to carefully align polymer molecular weight with the intended release profile.

End-group termination further modulates drug–polymer interactions and degradation behavior. This is highlighted by Ramos and collaborators, who evaluate an ISFI formulation for the treatment of periodontitis using ibuprofen and chlorhexidine as active ingredients. They used PLGA 50:50 with an acidic end group (trade name: Resomer RG 502H). Their results showed that the formulation released the drug in a controlled manner over several weeks, ensuring an antimicrobial effect [63]. A similar effect occurs in polymers with uncapped terminal groups compared to those protected with an aliphatic ester terminal group [64]. Consequently, end-group chemistry should be considered a critical CMA, particularly for drugs sensitive to rapid release or acidic microenvironments.

**Figure 6:**
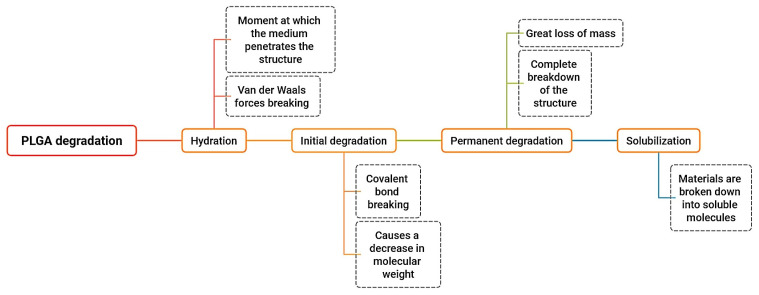
Phases of PLGA degradation. Colored boxes represent the main degradation stages, while dotted boxes indicate the associated physicochemical processes occurring during each stage.

PLGA degradation occurs through hydrolysis of ester bonds, followed by autocatalytic degradation, ultimately breaking down into lactic acid and glycolic acid [65]. These monomers enter the Krebs cycle, leading to the final products: water, CO_2_, and heat [66]. The degradation process is divided into four stages [13] (Figure 6).

Depending on the polymer ratio, the degradation time of PLGA in the body varies from days to months. Studies indicate that PLGA 50:50 degrades in approximately 50–60 days [67]. PLGA is FDA-approved [68,69], and also recognized by the European Medicines Agency (EMA) [70]. From a QbD perspective, degradation kinetics are not merely a material property but a determinant of critical quality attributes such as burst release intensity, sustained-release phase duration, and drug stability within the implant. Therefore, systematic control of PLGA characteristics is essential to ensure predictable performance and reproducibility of PLGA-based ISFI formulations.

These studies indicate that PLGA physicochemical attributes—particularly molecular weight, lactide-to-glycolide ratio, and end-group chemistry—constitute dominant CMAs governing ISFI performance. While higher molecular weight polymers are often associated with prolonged release, their influence on burst release is strongly modulated by polymer hydrophilicity and terminal functional groups, which dictate water uptake, degradation kinetics, and local microenvironmental pH. Notably, evidence suggests that end-group chemistry may exert a disproportionate impact during early implant formation compared to molecular weight alone. Within a QbD framework, this highlights the necessity of evaluating PLGA attributes as an interdependent set of variables rather than as isolated parameters, particularly when burst release control and reproducibility are critical quality objectives.

ISGs may rely on small-molecule matrix-forming agents rather than polymeric systems. Borneol [71], and camphor are hydrophobic small molecules that undergo phase transformation upon solvent exchange, leading to matrix formation suitable for localized drug delivery [72]. These systems do not depend on polymer chain entanglement but rather on the precipitation and solidification of the small-molecule matrix after solvent diffusion.

Cholesterol has been investigated as a lipidic matrix-forming agent in ISG systems for intra-periodontal delivery of doxycycline hyclate, demonstrating sustained release characteristics for antimicrobial agents [73]. In that study, cholesterol-based systems formed solid matrices after solvent exchange and provided sustained drug release profiles. The authors reported that formulation composition influenced gel formation behavior and drug release rate, demonstrating that cholesterol content modulates matrix characteristics and release kinetics. However, its high crystallinity may result in brittle matrices and potential variability in implant microstructure, making cholesterol content a critical material attribute (CMA) influencing CQAs such as mechanical integrity, porosity, and drug release kinetics.

Also, saturated fatty acids of varying chain lengths have been studied as matrix-forming agents for localized delivery applications [74]. Fatty acid chain length influenced matrix formation and drug release behavior, with longer alkyl chains associated with slower release rates. The study highlights the role of lipid composition in governing diffusion and matrix characteristics in ISG systems. From a QbD perspective, fatty acid chain length directly governs matrix densification rate, solvent exchange kinetics, and drug diffusion pathways, thus representing a key CMA impacting burst release and sustained release performance.

In 2021, Khaing et al. investigated the physicochemical properties, drug release behavior, and antimicrobial activity of a natural resin-based matrix for in situ gel (ISG) formation (rosin and propolis), incorporating vancomycin hydrochloride for periodontitis treatment. The formulation demonstrated suitable mechanical strength and enhanced drug release [75].

From a formulation perspective, these findings suggest that lipid type and molecular structure play a crucial role in solvent exchange dynamics, matrix densification, and drug diffusion pathways. Therefore, in a QbD context, lipid composition may be considered a critical material attribute (CMA) influencing critical quality attributes (CQAs) such as burst release and sustained release performance.

#### 2.3.3. Solvent-Related CMAs

**Figure 7:**
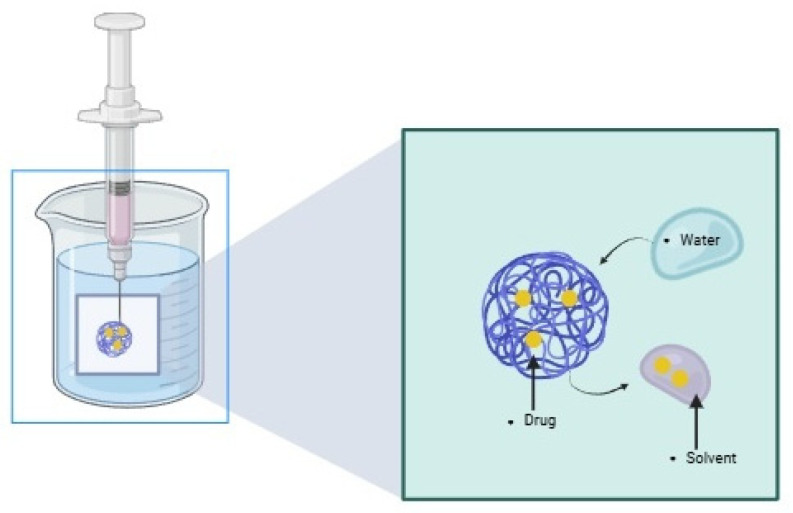
Solvent exchange process. Schematic representation of the solvent-exchange mechanism. Upon injection of the polymer–drug–solvent solution into an aqueous environment, water diffuses into the injected phase while the solvent diffuses outward, resulting in phase inversion and the formation of a solid or semi-solid implant depot. Created in BioRender. Campos, 2. (2025) https://BioRender.com/gs4w2j4, accessed on 10 December 2025.

Within a QbD framework, the solvent represents a CMA that directly governs implant formation, drug release kinetics, and local tolerability of ISFIs. Solvent selection determines the rate and extent of phase inversion upon injection, thereby influencing implant morphology, solvent exchange dynamics, and the magnitude of the initial burst release [57,76] (Figure 7).

Solvents used in ISFIs are typically characterized by their miscibility in water. Highly water-miscible solvents, such as N-methyl-2-pyrrolidone (NMP), dimethyl sulfoxide (DMSO), propylene glycol, acetone, and tetrahydrofuran, promote rapid water ingress and polymer precipitation, often resulting in faster implant solidification but increased burst release. In contrast, partially water-miscible solvents slow down phase inversion, leading to denser implant structures and more gradual drug release profiles, such as ethyl benzoate, ethyl acetate, triethyl citrate, and triacetin [77,78].

From a QbD perspective, the solvent diffusion rate is a key determinant of burst release intensity. Rapid solvent efflux during early implant formation facilitates drug migration into the surrounding medium, whereas reduced solvent exchange can mitigate this effect [79]. Consequently, solvent selection must be aligned with the target release profile defined in the quality target product profile (QTPP).

In addition to performance considerations, solvent-related toxicity represents a critical quality attribute associated with ISFI safety. Studies have shown that the myotoxicity ranking of these solvents, from highest to lowest, is: 2-pyrrolidone > DMSO > NMP [80].

To address these limitations, alternative strategies within a QbD framework include the use of less toxic solvents, solvent blends, or viscosity-modifying excipients to modulate solvent exchange without compromising injectability or release control. Some proposed solvents include ethanol, polyethylene glycol, and triethyl citrate [63]. Camargo and collaborators studied the effects of using different types of solvents in PLA-based ISFI formulations, with variations in their physicochemical properties, on the in vitro release of ivermectin. They evaluated NMP, 2-pyrrolidone, triacetin and benzyl benzoate. They concluded that the major factors affecting the drug release rate were the miscibility and viscosity of the polymer solutions, with the observed order being NMP > 2-pyrrolidone > triacetin > benzyl benzoate [76]. This study highlights the critical importance of solvent selection in ISFI formulations.

Solvent selection emerges as a high-impact CMA that directly governs phase inversion kinetics, implant morphology, and the extent of initial burst release in solvent-exchange ISFIs. Highly water-miscible solvents accelerate solvent efflux and polymer precipitation, often exacerbating burst release and local tissue exposure, whereas partially miscible solvents promote more gradual implant formation at the expense of injectability or formulation homogeneity. Beyond physicochemical performance, solvent-related toxicity and regulatory acceptability further constrain formulation design. From a QbD standpoint, solvent choice represents a critical trade-off between release control, safety, and translational feasibility, reinforcing the importance of risk-based solvent prioritization rather than empirical selection.

#### 2.3.4. Additive-Related CMAs

From a QbD perspective, additives are considered secondary CMAs used to fine-tune implant formation, internal structure, and drug-release behavior of ISFIs. Although not essential for implant formation, additives can significantly influence CQAs, particularly burst release, porosity, and mechanical integrity of the formed depot [81].

Hydrophilic additives, such as polyvinylpyrrolidone (PVP) [82], mannitol [83], or hydroxypropyl methylcellulose (HPMC) [84], are commonly incorporated to modify solvent exchange kinetics and implant porosity. Their presence can facilitate water penetration into the polymer matrix, altering phase inversion dynamics and, depending on concentration, either increasing or attenuating the initial burst release. In addition, certain hydrophilic polymers, such as HPMC, may enhance implant cohesion and local adhesion, which is particularly relevant for site-specific delivery [85].

Hydrophobic additives, including fatty acids such as stearic acid [81], have been explored to modulate wettability and reduce rapid solvent diffusion during early implant formation. Stearic acid decreases the wettability of a substrate by lowering its surface energy, creating a lubricant effect. It presents a hydrophobic tail layer to the medium by forming layered structures [86]. Molecules such as surfactants, which have amphiphilic properties, reduce surface tension in the phase inversion process, increasing permeation [87].

#### 2.3.5. Risk Management Strategies for CMAs

In PLGA-based solvent-exchange ISFIs, formulation composition plays a more dominant role in determining product quality than process-related parameters. As a result, CMAs associated with the polymer, drug, solvent, and additives represent the principal sources of risk within the system. To prioritize the relative impact of each CMA on product performance, a qualitative risk assessment approach can be employed. Table 4 presents an example of a risk matrix for PLGA-based ISFIs, in which CMAs are ranked according to their influence on key CQAs.

The risk matrix presented in Table 4 summarizes the relative impact of each formulation variable on key CQAs associated with ISFIs. For example, drug physicochemical properties (Drug) and polymer composition (Ratio L:G) typically exert a high influence on burst release and product degradation due to their impact on solvent exchange and polymer precipitation. This matrix provides a qualitative overview of how critical material attributes can affect ISFI performance and is intended to support prioritization during QbD-based risk assessment. This step enables the rational allocation of experimental resources and supports the development of robust, reproducible ISFI formulations.

### 2.4. Identify the Critical Process Parameters (CPPs) of ISFI

**Figure 8:**
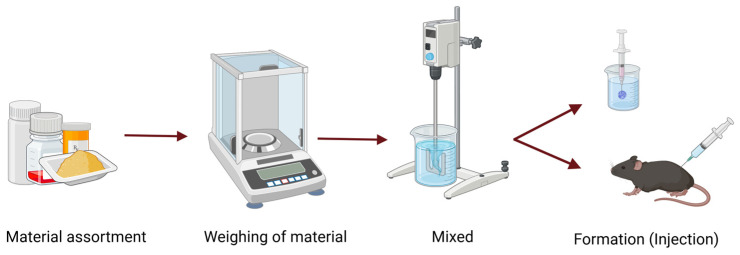
ISFI preparation process. Created in BioRender. Campos Campos, N. (2025) https://BioRender.com/ld9t2ts, accessed on 10 December 2025.

CPPs are identified based on their potential impact on critical quality attributes (CQAs) and their interaction with critical material attributes (CMAs) and can prevent deviations [88]. Kempe and colleagues studied the importance of monitoring ISFI formation during in vitro and in vivo administrations using electron paramagnetic resonance spectroscopy. Their results provide a better understanding of formation mechanisms in these systems, which is a key parameter in the implant development process [89]. However, as previously mentioned, the ISFI manufacturing process is relatively simple [90], as explained in greater detail in Figure 8.

For ISFIs formed by solvent-exchange mechanisms, the most relevant CPPs are associated with early-stage operations, including accurate material weighing and homogeneous mixing, as these steps directly influence formulation consistency and drug distribution. Implant formation itself—whether occurring in vitro or in vivo—represents a process-related step with potential impact on burst release; however, its influence is largely governed by formulation-dependent CMAs rather than by controllable processing conditions. The relative impact of CPPs on ISFI performance can be qualitatively assessed using risk-based tools. Table 5 illustrates an example of a risk matrix applied to the ISFI preparation process.

The risk matrix is useful at this stage for identifying the process parameters that will directly impact the CQA of the final product [91].

Another process that must be considered is the sterilization of formulations prior to administration. One approach to sterilizing ISFI formulations without compromising their physicochemical characteristics is aseptic filtration [92]. Korova and co-workers, in a formulation developed for HIV treatment, employed 0.2 µm filters for sterilization [93]. Similarly, Van Hemelryck et al. reported the use of sterile filtration through a 0.2 µm Whatman filter for in situ-forming gels (ISGs) and a 0.22 µm Sterivex filter for PEG 400–based solution formulations in a PLGA system containing bedaquiline fumarate salt [94].

CPP identification in ISFI development primarily serves to confirm process robustness and reproducibility, rather than as a major source of performance variability. This distinction is essential for guiding efficient development strategies and avoiding unnecessary process complexity.

### 2.5. Design of Experiments (DoE)

Design of Experiments (DoE) serves as a quantitative tool to systematically evaluate the relationships between critical material attributes (CMAs), critical process parameters (CPPs), and critical quality attributes (CQAs) [95]. Rather than functioning as an exploratory or empirical screening method, DoE enables risk-based decision making by identifying factor interactions, defining acceptable operating ranges, and supporting the establishment of a design space for ISFI formulations.

Factors:

- Polymer molecular weight.
- Polymer grade (in PLGA cases).
- The amount of polymer used.
- Presence/absence of additive.
- Additive characteristics.
- Miscibility of the solvent with water.
- Medium in which it is released.
- Forming method.

Response variables:

- Burst effect.
- The amount of drug released at different time points during treatment.
- Solvent exchange.
- Shape adopted by the implant.
- Effectiveness or bioactivity (antimicrobial activities, anticancer, anti-inflammatory).

Reported applications of DoE in ISFI development consistently demonstrate that formulation-related CMAs exert a greater influence on product performance than CPPs. Ibrahim and collaborators optimized an ISFI system for the antipsychotic risperidone using a Box–Behnken design (3^3^). The independent variables were lactide concentration (A), solvent type (B), and solvent/polymer ratio (C), while the response variables included formation time at 6 h, cumulative release over 40 days, and injectability time. The experimental design was created using Design-Expert software version 11 and included replicated center points, as well as points at the midpoints of each edge of the multidimensional cube to define the area of interest. Data were analyzed using analysis of variance (ANOVA, *p* < 0.05), along with R^2^, adjusted R^2^, predicted R^2^ values. Subsequently, 3D response and surface plots were generated. They reported that variables A and B showed negative effects on burst and cumulative release, whereas variable C denoted a positive effect. They also reported a negative action of the three variables on injectability time. The use of DoE allowed researchers to evaluate how independent variables influenced the dependent responses during ISFI development [96].

Kapoor and collaborators [97] optimized the formulation of ISFI for controlled delivery of an anti-HIV fusion inhibitor. The experiments were designed using experimental design software; they evaluated two factors (polymer:drug ratio and solvent type) at three levels (numeric and categorical). The response variable was the percent enfuvirtide release at 24 h and 48 h and viscosity. The results of the optimized formulation were validated for performance by preparing all three result formulations. This approach was satisfactory for extending protein release for a predetermined and specified duration of 48 h.

The primary value of DoE lies in its ability to support risk mitigation and rational optimization. By linking CMAs to CQAs through statistically validated models, DoE facilitates informed formulation adjustments and strengthens control strategies. Consequently, DoE represents a critical step in translating QbD principles into practical development outcomes for ISFI systems.

An example summarizing the critical variables and process parameters associated with an ISFI system is presented in Table 6.

In this example, the response variables correspond to the critical quality attributes (CQAs), namely initial burst release (%), drug release at 30 days (%), injectability force (N, measured using a texture analyzer), and viscosity (Pa·s).

In a typical optimization workflow, statistically significant factors are identified through ANOVA, followed by the generation of response surface and contour plots to visualize interactions between formulation variables. Subsequently, a composite desirability function is applied to simultaneously minimize burst release while maximizing sustained release performance and maintaining acceptable injectability.

The optimized formulation is then experimentally validated to confirm the predictive capability of the model. Common graphical tools used in ISFI optimization include 3D response surface plots, 2D contour plots, Pareto charts for factor significance analysis, and desirability function plots to define the optimal design space.

Optimization is subsequently performed using numerical desirability functions and graphical response surface analysis, allowing for the identification of operating regions that simultaneously satisfy multiple CQAs such as burst release, drug release at predefined time points, viscosity, and injectability force. The multidimensional combination of critical material attributes (CMAs) and critical process parameters (CPPs) that meet predefined QTPP criteria defines the design space [98]. This involves using predefined statistical criteria, including analysis of variance (ANOVA) with significance levels (*p* < 0.05) [99], evaluation of lack-of-fit, adjusted and predicted coefficients of determination, and residual diagnostics to ensure model reliability.

Using the experimental data reported by Kapoor et al. (2012) [97] response surface plots were constructed to illustrate how a QbD framework can define a multidimensional design space for ISFI systems. In the referenced study, viscosity was evaluated as a function of two formulation factors: polymer:drug ratio (1:1, 1.5:1, 2:1) and solvent type (DMSO, DMSO-triacetin 60:40, and benzyl benzoate and benzyl alcohol 70:30). These factors varied systematically, and viscosity (cP) was measured for each formulation.

**Figure 9:**
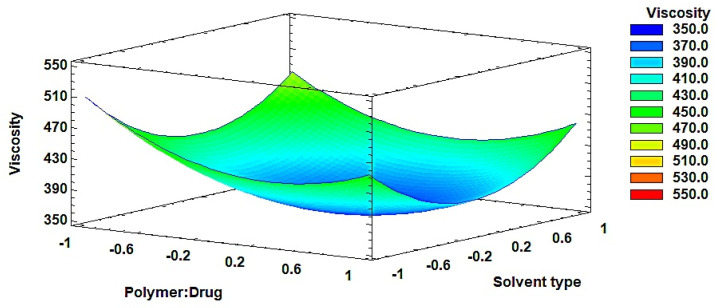
Response surface plot used to illustrate the multidimensional design space for viscosity in enfuvirtide ISFI formulations. Adapted from [97], with permission from Elsevier, 2012.

The resulting dataset was used to construct a response surface to visualize the combined effect of both variables and to identify a multidimensional region (design space) associated with acceptable viscosity. In Figure 9, the independent variables correspond to the polymer-to-drug ratio and the solvent type, both of which are known to influence formulation viscosity. The viscosity response surface demonstrates a clear dependence on both parameters. Increasing the polymer proportion from 1:1 to 2:1 resulted in a marked increase in viscosity, consistent with the higher polymer chain entanglement and solution density reported in the original study (352–532 cP). Furthermore, solvent selection significantly influenced viscosity, with formulations containing benzyl benzoate and benzyl alcohol exhibiting higher viscosity values compared to DMSO-based systems. The authors report that the design was evaluated by the response surface method using a polynomial equation. The equation for each response parameter was generated using ANOVA and multiple linear response analysis (MLRA).

The curvature observed in the response surface suggests an interaction between polymer concentration and solvent type, indicating that viscosity is governed by both polymer loading and solvent–polymer interactions. Within a QbD framework, viscosity represents a critical quality attribute (CQA) directly impacting injectability, manufacturability, and drug release. Higher viscosity formulations slow solvent exchange during implant formation, resulting in a denser polymer matrix and reduced pore formation. This structural change limits drug diffusion and decreases the initial burst release, leading to a more sustained drug release profile. Therefore, the design space can be defined as the region where polymer concentration and solvent selection provide acceptable viscosity while maintaining controlled drug release performance.

The authors reported that the experimental design was evaluated using response surface methodology (RSM) based on a polynomial equation. The equation describing each response parameter was generated using multiple linear regression analysis (MLRA), and the statistical significance of the model was evaluated by analysis of variance (ANOVA).

PLGA-based in situ-forming implants are mainly driven by material- and composition-related attributes. These include the type and physicochemical properties of the formulation components. In such systems, mixture designs, including simplex and I-optimal or D-optimal approaches, are particularly appropriate. This is because the experimental factors correspond to constrained component fractions whose total is fixed, allowing for a more robust definition of a composition-based design space. Nevertheless, the use of mixture design approaches is proposed as a rational and promising alternative to better capture the relationships between formulation variables and critical quality attributes.

## 3. Perspectives

The application of Quality by Design to in situ-forming implants has significantly advanced the rational development of these systems; however, several challenges continue to limit their broader translation and industrial implementation. Some of these limitations include irritation at the injection site, variability in the final shape of the implant [100], and the challenge of controlling the initial burst release [101]. Further research is also necessary, particularly in vivo studies, which should include a PLGA placebo group to properly assess any potential effects caused by the polymer itself within the body [102].

From a formulation perspective, expanding the application of QbD beyond traditional PLGA systems represents an important opportunity. Several alternative approaches could be explored to optimize and expand the use of these formulations, such as:

- Incorporating other types of systems, such as microparticles, liposomes, and microemulsions, to control drug release [103];
- Producing PLGA nanoparticles via the emulsion-evaporation method for drug encapsulation [104];
- Using ISFIs for brain administration, ensuring drug delivery directly to the central nervous system (CNS) [105]; and
- Leveraging ISFIs for a wide range of biomedical applications, making them a topic of significant interest in current research [106].

There are studies exploring their use in HIV treatment, such as the work by Benhabbour and colleagues, which highlights the significance of these types of formulations [107]. Similarly, there is interest in their use for parenteral administration of cannabidiol [108]. Jeganathan and collaborators optimized the release of chemotherapeutic agents and chemosensitizers through ISFIs aiming to reduce systemic toxicity and enhance drug retention at the target site [109]. A particularly innovative application of an ISFI involves its potential use in brain injury repair, where the system is loaded with nitric oxide to improve efficiency [110]. In this context, QbD-driven risk assessment tools will be essential to manage increased formulation complexity without compromising reproducibility. The versatility of ISFIs continues to drive innovation in controlled drug delivery. Although challenges such as burst release or solvent-related effects remain, ongoing research is focusing on improving formulation strategies to overcome these drawbacks.

Packaging is also a critical aspect of this type of system. Some commercial formulations employ Atrigel^®^ technology, which is based on prefilled syringe systems. These systems typically consist of two syringes: one containing the liquid phase and the other containing the powdered components. The syringes are connected through a specific coupling device that allows mixing of the formulation immediately prior to administration. After mixing, the syringes are disconnected and a needle is attached to enable injection. Commercial products based on this technology include Atridox^®^, Eligard^®^, Sublocade^®^, and Perseris^®^ [40].

Needle characteristics, such as length and gauge, must also be considered. For instance, Lupron Depot^®^ is administered using a dual-chamber syringe equipped with a 23 G needle, similar to Bydureon^®^. In contrast, Zoladex^®^ is administered using a larger needle, either 16 G or 14 G, depending on the dose. In the development of parenterally administered formulations, these factors are critical, as they directly influence syringeability and injectability, ultimately affecting the shape of the drug-eluting depot and, consequently, the drug release profile [111]. Nakmode and co-workers performed viscosity and injectability tests on an ISFI formulation containing levodopa and carbidopa for the treatment of Parkinson’s disease. They reported that the formulation exhibited Newtonian behavior, with a maximum injection force of 32.98 ± 0.72 N when administered using a 22 G needle [112].

The integration of advanced modeling approaches, such as mechanistic modeling and data-driven tools, offers promising perspectives for ISFI development. Coupling Design of Experiments with predictive models may enhance design space definition and reduce experimental burden. Overall, continued application of QbD principles, combined with critical evaluation of formulation and testing strategies, will be key to advancing ISFI technologies toward reliable and scalable long-acting drug delivery platforms.

**Figure 10:**
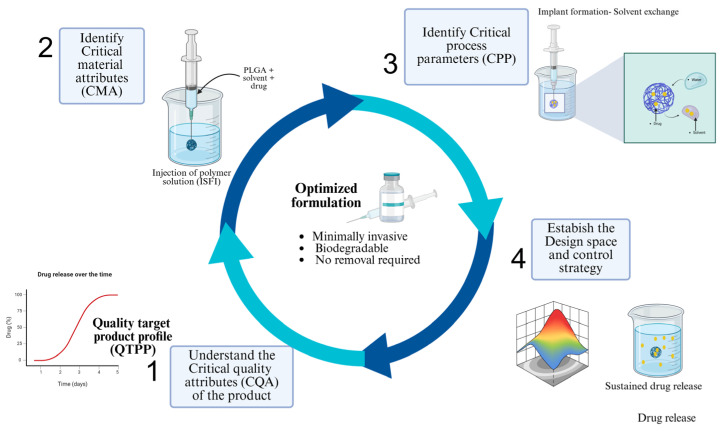
Schematic overview of the QbD approach applied to ISFI. Created in BioRender. Campos, 2. (2025) https://BioRender.com/41nj1uc, accessed on 10 December 2025.

The ISFI system involves a complex process; for this reason, Figure 10 provides a schematic overview of the key concepts discussed in this review. Figures and schematic illustrations were prepared using specialized graphical software; details are provided in Appendix A. The figure summarizes the steps involved in applying QbD to ISFI systems. This visual summary is intended to guide readers through the conceptual framework of QbD implementation in ISFI development, emphasizing the connection between process understanding and product performance. Model verification through confirmatory experimental runs at selected operating conditions supports scientific justification of the proposed design space. In alignment with ICH Q8 (R2) principles, this process extends to the establishment of a control strategy and lifecycle validation, including continued process verification to ensure consistent ISFI performance. This visual summary is therefore intended to guide readers through the conceptual framework of QbD implementation in ISFI development, emphasizing the direct connection between process understanding, statistical validation, optimization, and final product performance.

## 4. Conclusions

ISFIs are a pharmaceutical dosage form that offers numerous advantages over conventional implants. Their manufacturing process is relatively simple, requiring only a few excipients in their formulation. Despite some limitations, it is important to focus on improving the existing opportunities for optimization.

In this context, the application of Quality by Design provides a structured and risk-based framework to systematically address these limitations by linking formulation composition, material properties, and process understanding to critical quality attributes. This approach also helps reduce costs and development time, thus optimizing the design and the manufacturing process.

This review highlights that, in PLGA-based solvent-exchange ISFIs, product performance is predominantly governed by critical material attributes rather than by process-related parameters. Among these, polymer characteristics, drug physicochemical properties, and solvent selection play a central role in controlling implant formation kinetics, burst release, and long-term release profiles. Overall, the adoption of a QbD-driven approach offers a clear pathway to improve the design, optimization, and reproducibility of ISFI systems.

## Figures and Tables

**Table 1:** Quality target product profile (QTPP). Constructed based on the research of Patel and collaborators [25]. Fluorescein was used as a model drug to evaluate implant formation and drug release behavior.

Quality Objective	Value	Justification
Drug	Fluorescein (model drug)	Adjuvant therapy for post-ablative treatment
Doses	Dose required for therapeutic effect	Dose required to achieve the desired therapeutic effect
Design	In situ-forming implants	Extended-release systems to achieve the therapeutic effect
Evaluation subject	Model BD-IX rats	Animal study
Administration	Subcutaneous, necrotic tumor, non-necrotic tumor	Evaluation of drug release in different in vivo environments
Characteristics of the liquid formulation	Transparent solution with characteristic viscosity	All components must be dissolved or in suspension for administration and optimal viscosity
Characteristics of the formed implant	Amorphous solid (depending on the formation method) with the presence of pores	Formation of the matrix-based depot system that enables prolonged drug release
Physical properties of the implant	Using ultrasound and scanning electron microscopy methods	Knowing these parameters provides an understanding of the drug release
Release time	7 days	Duration of treatment

**Table 2:** Critical quality attributes: applied example. Constructed based on the research of Patel and collaborators [25].

Quality Attributes	Value	CQA	Justification
Appearance	Acceptable color and shape, the implant is not visible to the patient.	No	Does not directly affect safety or efficacy.
Size	Not visible to the patient	No	ISFI conforms to the shape of the injection site, so its size does not impact patient safety or efficacy.
Viscosity	Characteristic viscosity that allows subcutaneous administration	No	The formulation must have optimal viscosity for injection; it does not affect safety or efficacy.
Identification	USP 197, USP 621	Yes	Critical for safety and efficacy; must be controlled throughout the process.
Burst effect	A high initial release of drug and solvent	Yes	The initial release is a critical attribute, as a high release may cause myotoxicity or intoxication.
Assay	USP 621, USP 1225	Yes	It is a critical attribute because it directly impacts the stability of the drug.
Degradation products	Fluorescein monoglucuronide and fluorescein glucuronide	Yes	It is a critical attribute that impacts safety related to drug metabolism.

**Table 3:** Critical material attributes (CMAs) influencing the formulation, formation, and performance of PLGA-based in situ-forming implants (ISFIs).

CMA	Variables
Drug	Dose, molecular weight, solubility, molecular size
Polymer	Lactide:glycolide ratio, molecular weight, termination, chain length, viscosity
Solvent	Miscible in water
Additive	Solubility, viscosity

**Table 4:** Risk matrix of ISFIs. Color coding indicates the relative impact level of each factor on the corresponding CQA: red = high, yellow = medium, green = low.

CQA	Drug	L:G Ratio	MW Polymer	Polymer Termination	Solvent	Additive	Residual Solvent
Assay	High	Low	Medium	Medium	Medium	Medium	Low
Burst effect	High	High	Medium	Medium	Medium	Medium	Low
Solubility	Medium	High	High	High	High	Medium	Low
Product degradation	High	High	Medium	Medium	Medium	Low	Medium

**Table 5:** Risk matrix in the ISFI preparation process. Color coding indicates the relative impact level of each factor on the corresponding CQA: red = high, green = low.

CQA	Weigh	Mixed	Formation (Injection)
Assay	High	Low	Low
Burst effect	Low	Low	High
Solubility	Low	Low	Low
Product degradation	Low	Low	Low

**Table 6:** Representative DoE structure for PLGA-based ISFI development.

Factor (CMA/CPP)	Type	Low Level	Mid Level	High Level
PLGA concentration (%)	Numeric	30	40	50
Drug loading (% *w*/*w*)	Numeric	5	10	15
NMP proportion (%)	Numeric	40	50	60
Additive presence	Categorical	No	-	Yes

## Data Availability

Not applicable.

## Appendix A. Software Used for Figure Preparation

Figures included in this manuscript were prepared using BioRender (BioRender.com) for schematic illustrations. The figures were created under the BioRender academic license.
